# Quantification of Methane and Ammonia Emissions in a Naturally Ventilated Barn by Using Defined Criteria to Calculate Emission Rates

**DOI:** 10.3390/ani8050075

**Published:** 2018-05-16

**Authors:** Alexander J. Schmithausen, Inga Schiefler, Manfred Trimborn, Katrin Gerlach, Karl-Heinz Südekum, Martin Pries, Wolfgang Büscher

**Affiliations:** 1 Institute of Agricultural Engineering, University of Bonn, Nußallee 5, 53115 Bonn, Germany; m.trimborn@uni-bonn.de (M.T.); buescher@uni-bonn.de (W.B.); 2 Association for Bioeconomy Research (FBF), Adenauerallee 174, 53113 Bonn, Germany; si@fbf-forschung.de; 3 Institute of Animal Science, University of Bonn, Endenicher Allee 15, 53115 Bonn, Germany; kger@itw.uni-bonn.de (K.G.); ksue@itw.uni-bonn.de (K.-H.S.); 4 Chamber of Agriculture of North Rhine-Westphalia, Ostinghausen, 59505 Bad Sassendorf, Germany; martin.pries@lwk.nrw.de

**Keywords:** emission, dairy cows, greenhouse gas, tannin

## Abstract

**Simple Summary:**

Defined criteria for the application of the CO_2_ balance method in a naturally ventilated barn provided reliable data. This specification enabled the acquisition and quantification of CH_4_ and NH_3_ in a naturally ventilated dairy barn, as well as detecting decreasing NH_3_ emissions affected by supplementing an *Acacia mearnsii* condensed tannin extract to a dairy cattle ration. Moreover, long-term measurements were possible and can be used to examine feed-related mitigation strategies at a barn level in the future.

**Abstract:**

Extensive experimentation on individual animals in respiration chambers has already been carried out to evaluate the potential of dietary changes and opportunities to mitigate CH_4_ emissions from ruminants. Although it is difficult to determine the air exchange rate of open barn spaces, measurements at the herd level should provide similarly reliable and robust results. The primary objective of this study was (1) to define a validity range (data classification criteria (DCC)) for the variables of wind velocity and wind direction during long-term measurements at barn level; and (2) to apply this validity range to a feeding trial in a naturally cross-flow ventilated dairy barn. The application of the DCC permitted quantification of CH_4_ and NH_3_ emissions during a feeding trial consisting of four periods. Differences between the control group (no supplement) and the experimental group fed a ration supplemented with condensed *Acacia mearnsii* tannins (CT) became apparent. Notably, CT concentrations of 1% and 3% of ration dry matter did not reduce CH_4_ emissions. In contrast, NH_3_ emissions decreased 34.5% when 3% CT was supplemented. The data confirm that quantification of trace gases in a naturally ventilated barn at the herd level is possible.

## 1. Introduction

Environmental problems and emissions from agriculture are a topic with global significance and potential long-term consequences and are discussed by policy-makers and consumers [1,2]. Agriculture has to contribute to the mitigation of greenhouse gas (GHG) and NH_3_ emissions. In Europe, agriculture, in particular dairy farming, is the biggest source of NH_3_ emissions [3], which occur during slurry management but also directly out of the barn [4,5,6,7]. The potential of different mitigation measures, such as feeding strategies, on GHG emissions from naturally ventilated animal buildings is difficult to quantify [8]. Data available from the Intergovernmental Panel on Climate Change (IPCC) in particular are based on chamber measurements [9]. Here, data are mostly from theoretically calculated values or based on short-term measurements [10,11]. At the individual animal level, measurements of CH_4_ emissions from enteric fermentation in respiration chambers have been suitable to date [12]. The environmental conditions in a respiration chamber are strictly standardisable [9]. However, this method is cost- and labour-intensive and the natural behaviour of the animals is influenced [9,13,14]. Respiration chambers are rather unsuitable for the determination of NH_3_ emissions, because the influence of and interactions among animal activities, excrements, barn surfaces, weather effects and management are inadequately reflected in the measurements [4]. Further methods for measuring GHG in the natural environment of the animals include automated head chamber systems such as GreenFeed or the SF_6_ technique [15,16]. Such techniques are suitable for spot measurements of CH_4_ during pasture conditions or within the barn. However, especially for the GreenFeed system, it is a challenge to achieve data of all cows in a group of animals spread out over 24 h [16]. Furthermore, systems in conjunction with the individual animal neglect the GHG of the husbandry environment and excrements as well as additional trace gases such as NH_3_. More information on measurement systems are described by [11,16]. Therefore, measurements in the barn environment are necessary [17,18,19]. The determination of the ventilation rate (*VR*) in naturally ventilated buildings is a key factor for quantifying emissions from livestock buildings [20].

One of the methods routinely used to determine the *VR* in naturally ventilated barns is the tracer gas decay technique. For this procedure, several gases have been used: CO [21], ^85^Kr [22], N_2_O, and SF_6_ [23,24]. The *VR* can be calculated from the dilution of the dosed gases. It is difficult to realise a homogeneous distribution of the dosed trace gas [25], and SF_6_ is a GHG which is not produced by the animals in the barn, such as moisture, heat [20,22], or CO_2_ [26,27,28]. The CO_2_ balance method is the most commonly used. If the theoretical CO_2_ emission within the barn is known, based on the number of animals and individual animal characteristics (body weight (BW), lactation period), the *VR* can be quantified by reference to the actual CO_2_ concentration within the barn and consideration of the barn level [8,20,28]. 

Nevertheless, there is a lack of long-term measurements relative to selective short-term measurements or simulations [29,30]. Only a few studies have investigated the gaseous emissions from livestock buildings and their environmental implications based upon the CO_2_ balance method and defined measurement periods [4,17,31]. Little attention has been paid to the accuracy of long-term measurements including the corresponding basal, varying conditions. Long-term measurements involving feeding strategies for GHG mitigation under similar conditions (weather conditions, *VR*) are also still missing. Additionally, it is a challenge to adjust the methodology taking into account wind velocity and wind direction [31], which may largely impact the *VR*.

The main objectives of the present study were (1) to define a range of values for the CO_2_ balance method based on weather conditions, namely minimum wind velocity and the wind direction, which allow a reliable quantification of the *VR* in a specific naturally ventilated barn; (2) to quantify emission rates of CH_4_ and also NH_3_ on the herd level based on long-term measurements with robust and clearly defined values using the CO_2_ balance method combined with daily individual animal information such as dry matter intake (DMI); and (3) to evaluate CH_4_ and NH_3_ emissions in a naturally ventilated dairy barn by feeding a ration supplemented with selected secondary plant compounds under case-control conditions.

## 2. Materials and Methods

The study was conducted in a naturally ventilated dairy barn at the Experimental and Educational Centre for Agriculture ‘Haus Riswick’, Chamber of Agriculture of North Rhine-Westphalia, over a period of six months, during both the summer and winter seasons. The barn is located in Kleve, Germany (51°47′18′′ N, 6°8′19′′ E).

### 2.1. Experimental Building and Measurement Technique 

**Figure 1:**
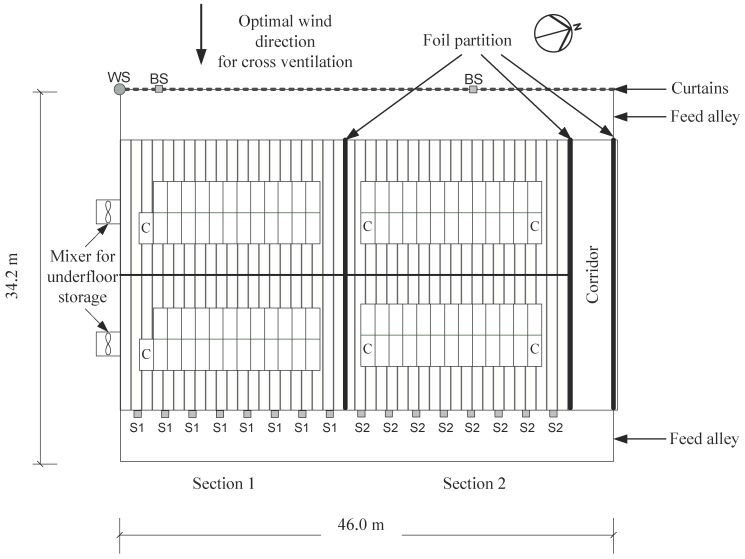
Construction plan of the barn and the slurry storage systems. Sections 1 and 2 have slatted floors. The level of slurry homogenisation varied among Sections 1 and 2. WS: weather station, located on the rooftop of the western eave; BS: background sampling points; S1–S2: sampling points (Sections 1 and 2) of the gases, described in the text; C: concentrate stations; small rectangles: slatted floor; wide rectangles: bedding area.

The dairy barn is a cross-flow ventilated building, with dimensions (length × width) of 68 m × 34 m. The eave height is 5.15 m and the ridge height is 12.35 m. The internal room volume of the barn is 21,000 m^3^. The ventilation was operated manually by adjusting the curtains on the western eave sides according to the prevailing weather conditions. The curtains were closed during the winter period, and in spring (10 March–16 April 2013) and summer (as from 16 April 2013), the curtains were half- or fully-opened. The building is spatially separated into three different sections with different air spaces. The measurements of this study were performed in Sections 1 and 2 (Figure 1), which were equipped with identical, slatted floors and an under-floor concrete slurry storage system (1.6 m depth). Sawdust was used as bedding material in the raised cubicles. The experimental barn was equipped with individual animal identification of water and feed intake as well as case control conditions of the gas measurements.

The slatted floor sections were cleaned four times per day using an automated cleaning robot with a water-spray device (Joz Tech JT100, Joz B.V., Westwoud, The Netherlands). Only the intensity of slurry homogenisation was slightly more intensive for Section 1 than Section 2 due to the mixer position at the gable wall of the building. The slurry was homogenised about ten times over the whole period. Because homogenisation of slurry increases emissions of trace gases, this period and a minimum of 12 h afterwards were not included in data analysis. Slurry was not removed during the experiment.

### 2.2. Measurement Concept 

Measurement of CO_2_, CH_4_, and NH_3_ concentrations to determine the CH_4_ and NH_3_ emissions were carried out in Sections 1 and 2 of the barn from 8 January to 25 June 2013, which included cold and warm periods. The overall experimental period was approximately 24 weeks (169 days) and was divided into four periods to examine different ration treatments (Table 1). 

The concentrations of NH_3_, CH_4_ and CO_2_ were measured continuously with a photoacoustic Multi-Gas Analyser 1412 and a Multiplexer 1303 (LumaSense Technologies SA, Ballerup, Denmark) via infrared spectroscopy as described by [32]. The accuracy of the Multi-Gas Analyser involved 2–3% absolute deviation of concentrations, and the detection limits given by the calibration chart of the manufacturer were as follows: 0.14 mg/m^3^ NH_3_, 0.27 mg/m^3^ CH_4_, and 9.34 mg/m^3^ CO_2_. 

During construction of the barn, gas measurement facilities were installed and the building was adjusted to be upstream of the prevailing western wind direction (210°–300°). Concentrations of CO_2_, CH_4_, and NH_3_ were measured and documented continuously. For the detection of the outlet air on the east side (lee) of the barn, eight sampling points were merged to one aggregate sample for each section (S1–S2; Figure 1). The background concentration was measured on the west side (windward) outside the barn (background sampling points; Figure 1). The sampling tubes were located at a height of 4 m above the feed alley and were equipped with filter orifices to protect them from dust. The air of each section was collected continuously by vacuum pumps (ME 2C, Vacuubrand GmbH + Co. KG, Wertheim, Germany) with a suction capacity of 33 L/min through a polytetrafluoroethylene tube in a sample bottle (600 mL). Every tube (8 mm inside diameter) was 100 m long, and the last 15 m of the tubes were laid underground to connect the barn with the service room where the equipment was located. This area and the service room were heated (heating cable, A. Rak Wärmetechnik GmbH, Frankfurt am Main, Germany) to minimise the influence of temperature and condensation in the tubes [4,32]. Consequently, the air exchange was calculated from a 600-mL sample bottle, which was flushed continuously. The whole tube system was flushed every 10 s. With a sampling interval of 240 to 300 s for each section, an actual and representative air sample could be ensured. Therefore, a sufficient flushing also for the adhesion of NH_3_ was ensured. The sampling bottles were flushed continuously (33 L/min), and the samples for analysis were taken after the described time intervals. The distance between the sampling bottle and gas analyser was as short as possible (less than 0.5 m). Additional information on the experimental setup is given in Schmithausen et al. [32].

Wind velocities, wind direction, air temperature, and humidity were measured outside the building with a weather station at a height of 6 m on the rooftop (north-west-corner) of the barn (Ahlborn Mess- und Regelungstechnik GmbH, Holzkirchen, Germany and LAMBRECHT meteo GmbH, Göttingen, Germany). Hence, during the whole measurement period, all weather conditions were recorded and the data were stored every four minutes (*n* = 57,930). The gas concentrations for each section were measured every five minutes (*n* = 48,672). To adjust weather conditions and gas concentrations, average hourly values were calculated (*n* = 3862).

The emission mass flow (M) was calculated using the gas concentration for both outlet (*C_outlet_*) and background (*C_background_*) air of each respective section. This was done according to Equation (1), whereas the *VR* of the barn was calculated using Equation (2).

(1) $$M=\;VR*\;(C_{outlet}-\;C_{background})$$

The *VR* was estimated by means of the CO_2_ balance method [26,27,28]. One heat production unit (HPU) was defined as equivalent to 1000 watts (W) of the total heat produced by the animals at 20 °C ambient temperature [26]. Concentration (*C*) is the difference between the background and outlet concentration of the examined gas.

(2) $$VR_{}=\;\frac{0.200}{\left(C_{outlet}-\;C_{background}\right)\;*\;10^{-6}}$$

At an individual animal level, the CO_2_ production rate (m^3^/h) per HPU during a medium feeding level is 0.185 [26,33]. Pedersen et al. [28] suggested a CO_2_ production rate per HPU of 0.200 m^3^/h in barns with indoor manure storage over a time period longer than three weeks. Thus, a production rate of 0.200 m^3^/h per HPU was selected. To determine the heat produced by one cow, the following formula was used ([26]; Equation (3)):

(3) $$\;H_{p\;}=5.6*BW^{0.75}+22*Y$$

where *H_p_* is the total heat production (*W*), *BW* is the body weight of the cow, and *Y* is the milk yield in kg per day.

### 2.3. Animals and Feeding

A feeding trial consisting of four experimental periods was conducted with high-yielding dairy cows. By supplementing a commercial product rich in condensed tannins (CT), an attempt was made to decrease methane emissions and improve the animals’ nitrogen use efficiency, as previously described by [34]. A total of 96 lactating German Holstein cows (25% primiparous, 112 days in milk (DIM)) were randomly allocated to one of two groups (control (CON) and experimental group (EXP)) and balanced for means of lactation number, DIM, milk yield, and BW. The animals were housed in Section 1 (CON) and Section 2 (EXP) of the barn. The 169-day feeding trail was divided into four periods, starting with a period (period 1) where both groups were fed the same ration without CT (CT0). During periods 2 and 3, the ration of the EXP was supplemented with 1% (CT1; period 2) and 3% of DM (CT3; period 3) of an extract rich in CT made from bark of *Acacia mearnsii* (declared concentration of CT 0.725 g/g DM, Weibull Black, TANAC S.A., Montenegro, Brazil) whereas the ration of CON was unchanged. During period 4, cows in both groups were fed the same ration (CT0) again (Table 1) [34]. 

An external rotary milking parlour in a neighbouring building was used to milk the cows twice a day (0500 h and 1630 h). Milking times were not included in the emission calculations.

The average daily milk yield at the beginning of the experiment was 36.6 ± 6.6 (kg/cow per day ± SD), and the cows had an average BW of 670 ± 63.3 (kg/cow). During the whole study, cows were fed a total mixed ration (TMR) consisting of grass silage, maize silage, sugar beet pulp, lucerne hay, concentrate (with CT in the EXP) and a mineral premix calculated for 37 kg of energy corrected milk (ECM). Rations were mixed once daily in a fodder mix wagon and distributed to and offered in computer-controlled weighing troughs (Waagen Döhrn GmbH & Co. KG, Wesel, Germany; RIC system, Insentec B.V., Marknesse, The Netherlands) for ad libitum intake. The cows were equipped with transponders on collars that allowed them to be identified at each visit and thus enabled the recording of individual daily feed and water intakes.

The DM of the mixed ration, DMI, milk yield (measured by the milking system), and BW as well as several other variables were measured daily; detailed information about the impact of the CT extract on DMI, animal productivity, nitrogen use efficiency and digestibility of rations is reported by Gerlach et al. [34].

### 2.4. Data Presentation

Data presentation of barn-level, i.e., group measurements, was carried out purely descriptively because the overall goal was to establish a general frame applying the two variables wind velocity and wind direction. A valid inclusion of more specific weather conditions and sound statistical evaluation would require all-season measurements which were outside the scope of this study. However, we think that the approach of defining a validity range called data classification criteria (DCC) for the variables of wind velocity and wind direction during long-term measurements at barn level provides a valid basis for continuing experimental studies. 

## 3. Results

### 3.1. Methodical Consideration

**Figure 2:**
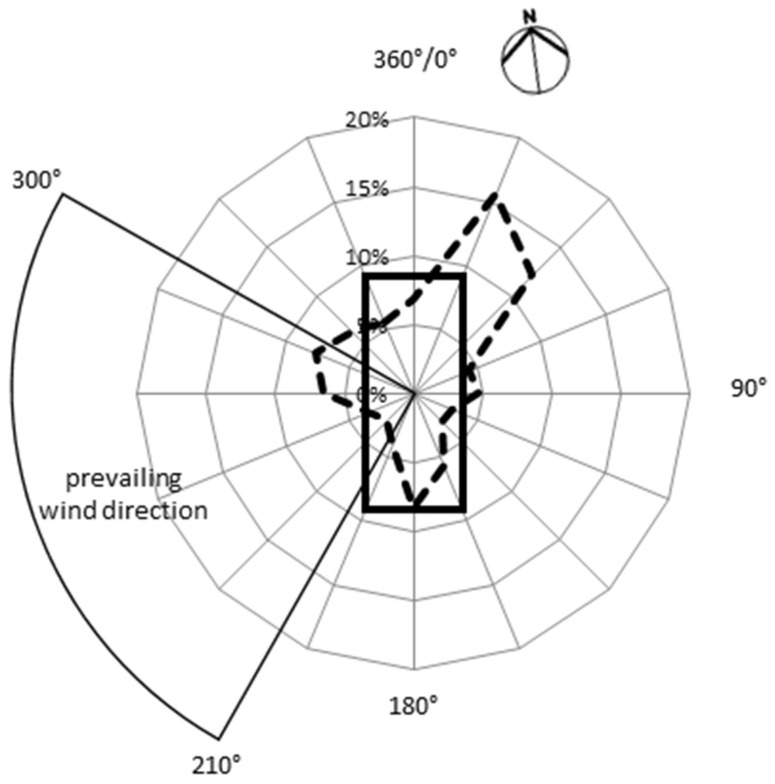
Plot of a wind rose with the selected angle of the upstream flow (210°–300°) specific for this barn building to realise cross flow ventilation (solid line: alignment of the barn to the prevailing wind direction; dashed line: frequency of the overall wind direction and a minimum wind velocity (≥0.7 m/s) per hour over the whole study (*n* = 3395)). Compass direction north (N) ≙ 345°.

To obtain representative data specifically for this barn building, only values consistent with the defined DCC were used. These criteria relate to topographical characteristics and architectural adjustment of dairy barns on the farmyard. Therefore, the prevailing angle of wind direction was 210° to 300° to realise cross flow ventilation for the building (Figure 2). The weather station on the barn roof provided usable data at wind velocities greater or equal to 0.7 m/s (detection limit) (*n* = 3327). Thus, for calculation of each wind direction $(\bar{D}$), only values ($D_{i}$) with a wind velocity $(V_{i}$) ≥ 0.7 m/s were used. To ensure that this average value was not based on individual gusts of wind, the average value was calculated from four or more single values per hour. Furthermore, only wind data with an hourly mean $(\bar{D}$) coming from sectors 210° to 300° were used (*n* = 710). Figure 2 shows the frequency distribution of wind direction during the experimental period as hourly means distributed in 16 sectors of 22.5°, respectively. Fewer than one-quarter of the values lay between 210° and 300°.

### 3.2. Calculation of the Ventilation Rate

The level of aggregation for the *VR* was from hourly values which were from a minimum of four individual values of wind speed and suitable wind direction per hour and then averaged to weekly means, because several days occurred in which no data could be measured that fulfilled the DCC. One day equals hours per day minus the milking times in the morning and afternoon; thus, a maximum of 18 h per day was used. Only data without any further herd management intervention were used for analyses.

**Figure 3:**
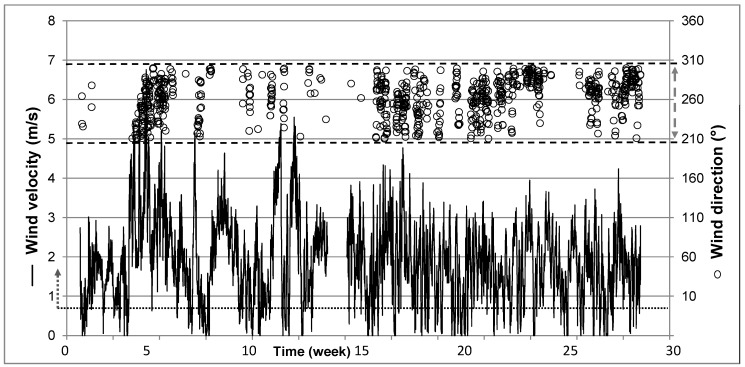
Time axis over 24 weeks (starting point x-axis 1 January 2013) with hourly mean values (*n* = 4032) of wind velocity (≥0.7 m/s; solid line; *n* = 3395) in the lower part and wind direction (ο, open circle) between 210° and 300° (*n* = 710) in the upper part of the Figure.

Figure 3 depicts the wind velocity and the wind direction over the whole experimental period of 24 weeks. Only 18% of the overall one-hour values (*n* = 3862) were valid according to the DCC. These hourly means are based on more than four consecutive values. Figure 3 also shows that the frequencies of reliable values during the 24-week period are irregularly distributed. Even longer periods occurred, such as from week 2 to 4 (14–27 January), when the wind velocity was permanently too low or the wind came from the wrong direction to allow reliable measurements.

**Figure 4:**
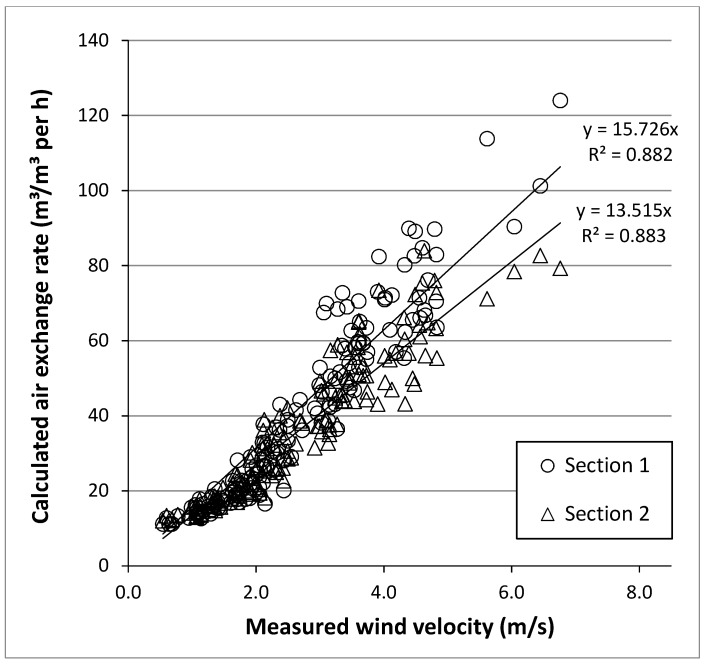
Relationship between the wind velocity (m/s) and the air exchange rate (m^3^/m^3^ per h) in the control group (CON) (Section 1: SE = 0.232) and experimental group (EXP) (Section 2: SE = 0.176) in the winter (*n* = 149) calculated with the CO_2_ balance method. The point of intersection was set through zero, and the wind velocity on the x-axis starts at 0.7 m/s.

The air exchange rates (m^3^/m^3^ per h) of Sections 1 and 2 in comparison with the measured wind velocity (m/s) at the barn are presented in Figure 4. Only measurements with closed curtains were considered. A simple regression of the calculated air exchange rate against the wind velocity resulted in an R^2^ of 0.882 for Section 1 and 0.883 for Section 2. The regression lines clarify the coherence. The initial equation of Section 1 (y = 18.3x − 8.5) had an intercept that was different from zero and, for the ease of comparison, the equation was recalculated and set through the zero point. The equation from Section 2 (y = 13.8x − 0.86) had an intercept that was not different from zero. Similarly, the slope of the line of Section 1 was greater such that, with a wind velocity of 4 m/s, a *VR* of 62.9 m^3^/m^3^ per hour was observed compared to 54.1 m^3^/m^3^ per hour for Section 2. Open curtains at similar wind velocity resulted in higher air exchange rates in the barn (unpublished observations, this study). 

### 3.3. Effect of Weather Conditions

**Figure 5:**
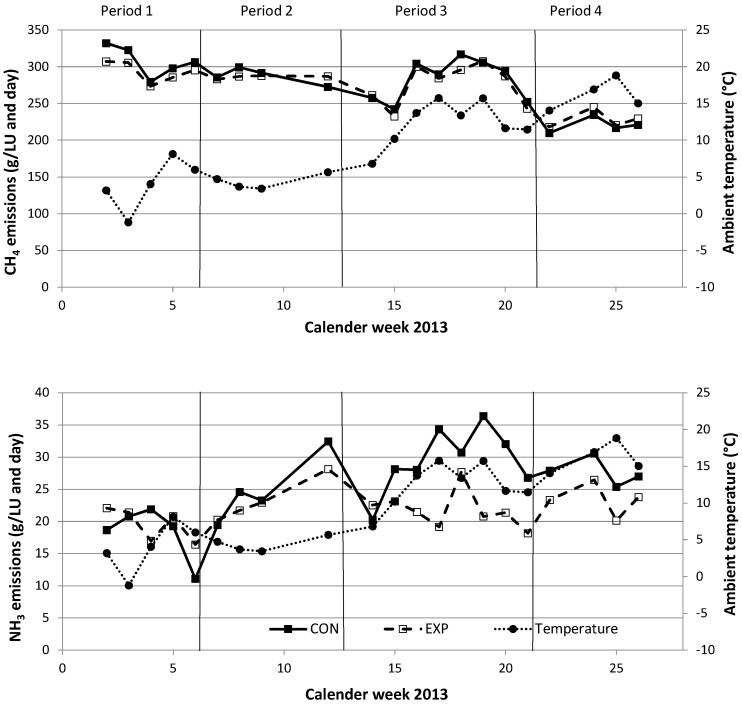
Methane and ammonia emissions of the control (CON; ■) and experimental (EXP; □) groups during four investigation periods (subdivided by vertical bars) with different ambient temperatures (●). Different feed rations are marked with a vertical bar. Data points show weekly means calculated from hourly means (see Section 3.2).

The variations in temperature, CH_4_ and NH_3_ emissions over calendar weeks 2 to 25 of 2013 are shown in Figure 5. The CH_4_ emission rates of both feeding groups decreased, whereas the temperature increased over the course of the measurement periods (4.4 °C to 15.2 °C; Table 1). In contrast, NH_3_ emissions from CON increased with increasing temperature from period 1 (18.4 g/LU per day) to period 4 (27.4 g/LU per day) (LU = livestock unit ≙ 500 kg BW), whereas NH_3_ emissions from EXP were nearly constant from period 2 (23.0 g/LU per day) to period 4 (22.2 g/LU per day; Table 2). 

### 3.4. Methane and Ammonia Emission Rates Related to Floor Section and Feeding Periods

Average emission rates of CH_4_ and NH_3_ per LU over four measurement periods are shown in Table 2. No difference was observed between EXP and CON rations for CH_4_ emission rates. There was no difference in NH_3_ emission rates between EXP and CON in periods 1 and 2. In contrast, NH_3_ emissions from CON, a difference to EXP occurred in period 3 and 4 (Table 2). In period 3, the feeding of tannins (CT3) in EXP decreased NH_3_ emissions by 34.5%. In period 4 (CT0), NH_3_ emissions were still lower for EXP cows by 23.4% (Table 2). Although all cows received the same ration during periods 1 and 4, in period 1, CH_4_ emissions of CON cows were lower and NH_3_ emissions were higher compared to EXP. In period 4, the contrary was observed: lower CH_4_ emissions of CON and higher emissions of NH_3_. 

## 4. Discussion

### 4.1. Methodical Considerations and Calculation of the Ventilation Rate

Models and databases to evaluate GHG emissions vary widely and are often not clearly defined. For example, Wu et al. [4] reported the effects of climatic factors and the air exchange rate on GHG emissions, but did not define any rules or measurement criteria for data validation. Using the CO_2_ balance method for measuring the *VR* [26,27], total emissions from a defined area (dairy barn) can be monitored. Management procedures in a barn can affect measuring emission rates compared to performance characteristics of the animals. For example, floor types, deep litter bedding systems, and indoor manure storage are additional sources of CO_2_ that must be included in balance calculations [27,33]. Consequently, established criteria (wind velocity and direction) for a valid range of measurement conditions are essential. In the literature, for example, the reference of continuous long-term measurements vary between measuring periods from some days [18] to several weeks [22,30,35] or even from four months [17] to 19 months [6]. 

In this study, long-term measurements (169 days) of CH_4_ and NH_3_ emission rates calculated by the CO_2_ balance method [26,27,28] enabled measurements at a practical scale in a dairy barn. Effects such as outgassing from the excrements during the milking times when the cows were outside the barn has been considered. Furthermore, the method allowed adjustments for different variables such as the BW of the cows and the milk yield [4], such that changes in feeding level and their effects could be estimated. Furthermore, Wu et al. [4] favoured the CO_2_ balance method over the tracer gas decay method for the estimation of gas emissions, considering that the cows provide a good mix of CO_2_ in the barn air. Subsequently, the produced CO_2_ is an optimal point of reference. Additionally, the DMI between Sections 1 and 2 was similar in this experiment [34], such that not only was the ration well defined, but also the CO_2_ production was similar.

In this study, the selected barn allowed for the measurement of GHG emissions of two feeding groups (EXP and CON) within the same barn simultaneously (case-control conditions). The measurement frequency (every four minutes at the same measuring point; 15 values per hour) provides an accurate assessment in the differences between the feeding groups, and reduces feasible inaccuracy in the measurement technique.

One major result of this study was that the DCC have a significant influence on the validity of the emission rate estimates of the whole barn. The established characteristics of the DCC show that some periods of measured data (Figure 3) are unsuitable for a naturally ventilated barn with a prevailing wind direction. The findings confirm that measurements in naturally ventilated dairy barns should be carried out for as long as necessary in order to obtain a sufficient number of data points without the confounding influence of, for example, weather conditions. Therefore, short-term or spot measurements are more inconclusive than long-term investigations. In this study, only about 12% of averaged emission data (CH_4_ and NH_3_) were categorised as reliable and accurate when judged by wind velocity and wind direction as established by the DCC. As another result of this study, the measurement conditions within both sections of the barn are congruent, which guaranteed equally robust and reliable data for groups of cows receiving treatments that differ between barn sections. The relationship between wind velocity and air exchange rate demonstrated an accurate coherence between both groups (Figure 4). Small differences in air exchange rate (8.8 m^3^/m^3^ per h) between Sections 1 and 2 are regularly occurring and are probably effected by thermal events. Such differences can vary in response to curtain position and effect of wind vector. Therefore, it is recommended to apply key variables (DCC) in different types of buildings to derive building-specific data using the CO_2_ balance method under case-control conditions and to get more reliable data that may eventually serve as a standard for gaseous emissions from livestock facilities. 

### 4.2. Effect of Weather Conditions

Weather conditions have a large impact on GHG emissions. Ammonia emissions increase with increasing temperature [33,35]. Rong et al. [35] observed a range between 4.53 g NH_3_/HPU per day (ambient: 7.8 °C) and 17.72 g NH_3_/HPU per day (ambient: 19.9 °C). In this study, the NH_3_ emission rate of CON cows increased from period 1 to period 3 concurrently with the average temperature. In contrast, the NH_3_ emission rate of the EXP cows was unaffected by temperature. Thus, it was assumed that the EXP ration reduced NH_3_ emissions compared to the CON. In contrast, no effect was observed of the temperature on CH_4_ emissions. In contrast, Pereira et al. [36] observed a significant increase in CH_4_ production from dairy cattle excreta with change in storage temperature from 5 to 35 °C. Zhang et al. [33] also reported a greater correlation between temperature and NH_3_ emissions than of temperature and CH_4_ emissions. Still, it is recommended to measure simultaneously weather conditions for the experimental area and the climate conditions in an experimental building or on commercial farms and especially to distinguish between the storage areas of the slurry. Storage underneath the slatted floor within a building is much less influenced by ambient atmosphere than storage outside under varying and shifting weather conditions [37].

### 4.3. Methane and Ammonia Emission Rates Related to Floor Section and Feeding Periods

The calculated CH_4_ emission rates were similar to previous studies [8,17,35]. However, the present study only focused on those hours per day (maximum of *n* = 18) without the potential influence on the data of management (e.g., feeding and milking time, slurry homogenisation, cleaning of the walking alleys and bedding areas). Data obtained during these time periods were excluded, because the dairy cows which served as a basis for the CO_2_ balance method were outside the barn. The CH_4_ emission rate was similar to that found by Cortus et al. [6], who reported an average CH_4_ emission of 290 g/animal unit (≙464 kg BW) per day during 20 months of sampling in dairy barns with solid floors. 

In this study, no differences in the CH_4_ emission rate between EXP and CON were found in any periods. These results disagree with other studies: Grainger et al. [38] using the tracer gas technique, reported a reduction in the range of 10 to 22% (g CH_4_/kg DMI) by supplementing 0.9% and 1.8% of DMI of *Acacia mearnsii* CT to the ration of grazing dairy cows. Measurements taken over some days where higher concentrations of tanniferous extracts in rations were applied decreased CH_4_ emissions [39,40]. In a comprehensive review of mitigation options, Hristov et al. [40] reported a low CH_4_ mitigation effect (<10%) of CT and noted a lack of data regarding long-term studies on effects of CT.

The NH_3_ emissions across all periods (18.4–30.0 g NH_3_/LU per day) were higher than those reported by Rong et al. [35]. Although there was no effect on NH_3_ emissions when 1% CT extract was fed (period 2; CT1), feeding 3% CT in the ration (period 3; CT3) decreased the NH_3_ emission rate by 34.5%. However, in comparison with period 1, during period 4 (CT0) the NH_3_ emissions in CON were still higher. Furthermore, they were only 8.6% lower than in period 3. This indicates that the mitigation effect was persistent during the feeding periods and lasted until period 4, when all cows were again fed the same ration. In period 4, the animal-based variables such as N in milk decreased much faster than the NH_3_ emissions within the barn [34]. Probably, in addition to the temperature effect, the effect of longer-lasting NH_3_ emissions was caused by slurry stored below the slatted floor in the EXP and CON groups. Grainger et al. [38] reported a shift in excreted N from urine to faeces as a response to CT supplementation. Urea, the soluble and rapidly degradable N compound in urine, causes a fast release of NH_3_. Vice versa, when, at the same N intake, excreted N is shifted to faeces, continuous emissions from the surface crust of the stored slurry will increase [29,41]. 

Compared with the statements of Montes et al. [41], the overall emission rates of NH_3_ for the EXP and CON groups can also, in part, be attributed to the permanent under floor storage of slurry [41]. The focus of this study was to characterise the mitigation potential and not the absolute emission rates based on the comparison of two feeding groups and their emissions within the same building. 

## 5. Conclusions

The study describes defined criteria for calculating the emission rates of a naturally ventilated dairy barn housed with dairy cows in Germany. To date, little attention has been paid to the accuracy of long-term measurements including the corresponding varying basal conditions. The experiment made it possible to quantify gaseous emissions on a herd level, and the results demonstrate that the photoacoustic system is suitable for measuring CH_4_ and NH_3_ for long-term measurements in dairy barns. The amount of reliable, utilisable data showed that a definition of the length of measurement periods is indispensable. The applied criteria are unique for each building and need to be considered on different barns in further research. The results of the emission values showed mitigation effects of NH_3_ (34.5%) during feeding 3% of DM of condensed tannins (*Acacia mearnsii*) in the ration on herd level, whereas CH_4_ was not affected. Therefore, such long-term experiments on a practical scale provide more information on the long-term effects of feed additives for ruminants.

## Figures and Tables

**Table 1:** Description of the measurement periods and conditions (average values of each measurement period for both barn sections). DM: dry matter.

Period	Time of the Measurement Period	No. of Days	Concentration of Tannins (% of DM)	Temperature ^1^ during Measure °C (SD)	Wind Speed ^2^ during Measure m/s (SD)
Period 1 (CT0)	8 January 2013–4 February 2013	28	0	4.4 (3.9)	2.2 (1.1)
Period 2 (CT1)	5 February 2013–18 March 2013	42	1	4.9 (2.7)	1.7 (0.6)
Period 3 (CT3)	19 March 2013–20 May 2013	63	3	12.5 (3.0)	1.7 (0.5)
Period 4 (CT0)	21 May 2013–25 June 2013	36	0	15.2 (2.9)	2.0 (0.1)

^1^ Temperature inside the naturally ventilated barn; ^2^ Wind speed measured at the weather station outside the barn building.

**Table 2:** Barn level CH_4_ and NH_3_ emissions averaged over the feeding periods. GHG: greenhouse gases.

Item	GHG in Corresponding Period					
	Methane Emission			Ammonia Emission		
Period	Control group (CON)	Experimental group (EXP)	CON/EXP (%)	Control group (CON)	Experimental group (EXP)	CON/EXP (%)
Period 1 (CT0)						
g/LU per day (SD)	307.1 (20.9)	291.9 (14.3)	−5.2	18.4 (4.1)	19.8 (2.3)	+7.1
kg/LU per year	112.1	106.5		6.7	7.2	
Period 2 (CT1)						
g/LU per day (SD)	290.6 (24.4)	289.2 (7.9)	−0.5	24.2 (9.6)	23.0 (6.0)	−5.2
kg/LU per year	106.1	105.6		8.9	8.4	
Period 3 (CT3)						
g/LU per day (SD)	289.6 (26.1)	282.8 (24.7)	−2.4	30.0 (4.9)	22.3 (2.5)	−34.5
kg/LU per year	105.7	103.2		10.9	8.1	
Period 4 (CT0)						
g/LU per day (SD)	225.1 (13.8)	229.6 (11.0)	+2.0	27.4 (2.0)	22.2 (3.5)	−23.4
kg/LU per year	82.2	83.8		10.0	8.1	

LU = livestock unit ≙ 500 kg body weight; CT0 = all groups were fed without supplemented condensed tannins in the ration; CT1 = experimental group was fed with 1% condensed tannins in the ration; CT3 = experimental group was fed with 3% condensed tannins in the ration.
